# Efficacy of Oral Ginger (*Zingiber officinale*) for Dysmenorrhea: A Systematic Review and Meta-Analysis

**DOI:** 10.1155/2016/6295737

**Published:** 2016-05-05

**Authors:** Chen X. Chen, Bruce Barrett, Kristine L. Kwekkeboom

**Affiliations:** ^1^Indiana University School of Nursing, Indianapolis, IN 46202, USA; ^2^Department of Family Medicine and Community Health, University of Wisconsin-Madison, Madison, WI 53715, USA; ^3^School of Nursing, University of Wisconsin-Madison, Madison, WI 53705, USA

## Abstract

This systematic review examines the efficacy of oral ginger for dysmenorrhea. Key biomedical databases and grey literature were searched. We included randomized controlled trials comparing oral ginger against placebo or active treatment in women with dysmenorrhea. Six trials were identified. Two authors independently reviewed the articles, extracted data, and assessed risk of bias. Discrepancies were resolved by consensus with a third reviewer. We completed a narrative synthesis of all six studies and exploratory meta-analyses of three studies comparing ginger with placebo and two studies comparing ginger with a nonsteroidal anti-inflammatory drug (NSAID). Ginger appeared more effective for reducing pain severity than placebo. The weighted mean difference on a 10 cm visual analogue scale was 1.55 cm (favoring ginger) (95% CI 0.68 to 2.43). No significant difference was found between ginger and mefenamic acid (an NSAID). The standardized mean difference was 0 (95% CI −0.40 to 0.41). Available data suggest that oral ginger could be an effective treatment for menstrual pain in dysmenorrhea. Findings, however, need to be interpreted with caution because of the small number of studies, poor methodological quality of the studies, and high heterogeneity across trials. The review highlights the need for future trials with high methodological quality.

## 1. Introduction

Dysmenorrhea is characterized by low abdominal or pelvic pain occurring before or during menstruation [1]. It can be either primary (in the absence of an identifiable pathological condition) or secondary (due to organic pathology such as endometriosis or fibroids) [1]. In addition to pelvic pain, some women with dysmenorrhea may also experience low back pain, nausea, vomiting, and changes in bowel habits [1, 2]. Dysmenorrhea is highly prevalent among women of reproductive age, with an estimated prevalence between 16% and 91% [3]. As the leading cause of absenteeism from work or school among women [4], dysmenorrhea negatively affects individual women and society as a whole. The impact of dysmenorrhea can even extend beyond the time of menstruation in that dysmenorrhea leads to increased pain sensitivity among affected women [5, 6]. Such increased pain sensitivity and other shared mechanisms of pain (e.g., inflammation) may explain why dysmenorrhea commonly cooccurs with other chronic pain conditions (e.g., irritable bowel syndrome, low back pain, and chronic headache) [6]. Scholars suggest that such increased pain sensitivity may predispose women to developing pain later in life [6]. Better management of dysmenorrhea may not only improve women's quality of life, but also reduce their risk of developing future pain [5, 6].

Dysmenorrhea is conventionally treated with nonsteroidal anti-inflammatory drugs (NSAIDs) or oral contraceptive pills (OCPs) [2], the efficacy of which are supported by research evidence [7, 8]. However, NSAIDs and OCPs have limitations: some women with dysmenorrhea do not respond to NSAIDs or OCPs (with an estimated failure rate of >15% for NSAIDs) [2, 4]; some cannot use these medications because of contraindications or adverse effects; some prefer not to use any medications. Therefore, investigation of complementary alternative treatments for dysmenorrhea is warranted. With a long history of medicinal use that dates back over 2,500 years, ginger rhizome has played an important role in Chinese and Indian medicine [9]. Oral ginger has been used to treat pain from dysmenorrhea, osteoarthritis, rheumatoid arthritis, and migraine, as well as gastrointestinal symptoms such as nausea, vomiting, diarrhea, and indigestion [9]. Ginger is one of the most commonly used natural products among women with dysmenorrhea [10]. The exact mechanism of action of ginger in pain relief remains to be elucidated; however, some evidence suggests that the constituents of ginger have anti-inflammatory and analgesic properties [11]. Furthermore, preclinical research shows that ginger suppresses the synthesis of prostaglandin (through inhibition of cyclooxygenase) and leukotrienes [9], which are involved in dysmenorrhea pathogenesis [2].

Two systematic reviews examined the efficacy of ginger for general pain relief (acute and chronic pain) [12, 13] and included the few early trials specific to ginger in dysmenorrhea [14, 15]. During finalization of our paper, a review of ginger for primary dysmenorrhea was published [16], but findings were limited by several factors including potential author bias from conflict of financial interest, a less stringent evaluation of study bias, problematic selection of trial data for effect size calculations, and exclusion of comparisons of ginger with NSAIDs (widely used in dysmenorrhea management) in meta-analysis. Thus, a more rigorous and thorough systematic review and meta-analysis is merited. The purpose of this review was to systematically summarize and critically evaluate the evidence from clinical trials of oral ginger for the treatment of dysmenorrhea. The research question was as follows: In women with dysmenorrhea (population/problem), is oral ginger (intervention) as compared to placebo control or other interventions (comparison) efficacious in reducing menstrual pain (primary outcome)?

## 2. Methods

A prospective protocol was developed following the Cochrane Handbook (http://handbook.cochrane.org/) [17]. It included a description of the research question, search strategy, inclusion criteria, data extraction, and evaluation criteria. The protocol was registered on the international prospective register of systematic reviews PROSPERO (CRD42015016744) and can be accessed at http://www.crd.york.ac.uk/PROSPERO/display_record.asp?ID=CRD42015016744. We followed the Preferred Reporting Items for Systematic Review and Meta-Analysis (PRISMA) statement [18] for the reporting structure of this review.

### 2.1. Search Strategies

We searched the following electronic databases: PubMed, EMBASE, Cochrane Library, Cumulative Index to Nursing and Allied Health Literature (CINAHL), Web of Science Core Collection (including Science Citation Index, Social Science Citation Index, and Conference Proceedings Citation Index), PsycINFO, the Allied and Complementary Medicine Database (AMED), LILACS, International Pharmaceutical Abstracts, and Biological Abstracts. Websites of clinical trial registries (including ClinicalTrial.gov, World Health Organization International Clinical Trial Registry Platform) were searched to identify unpublished trial data. We also searched grey literature (i.e., research that is not formally published in journal articles) through Open Grey and Grey Literature Report. Unpublished trial data and grey literature were searched to minimize potential publication bias [17].

The search strategy was (ginger^*∗*^ OR zingiber^*∗*^ OR zinziber OR gingifere OR gingembre OR Jiang OR shokyo) AND (dysmenor^*∗*^ OR menstr^*∗*^ OR “period^*∗*^ pain” OR “painful period^*∗*^”). Words were searched as free text. Following the Cochrane Handbook [17], no restrictions in language or publication date were applied. Bilingual colleagues were sought to assist with translating non-English publications. All the databases and websites were searched from their respective inceptions to May 2015. Reference lists of the retrieved articles and previous reviews were hand-searched to identify relevant studies.

### 2.2. Study Selection

We included those trials in which oral ginger was used as a primary, sole, and not a combined therapy and compared against a placebo or active treatment in women with dysmenorrhea. We included only randomized controlled trials reporting menstrual pain severity assessed by a patient-reported outcome measure (e.g., visual analogue scale or verbal rating scale). The exclusion criteria were (1) trials of ginger combined with other potentially active substances, (2) trials of nonoral use of ginger (e.g., ginger moxibustion, essential oil massage), (3) nonhuman or in vitro studies, and (4) observational studies.

All three authors were involved in reviewing and extracting data. Two reviewers extracted data from the included trials independently: first author (CC) reviewed all studies; the second and third authors (BB and KK) each reviewed half. Disagreements were resolved by discussion and consensus with assistance from the third reviewer. A standard data-coding table was developed for extracting data from individual trials. The following data were extracted: participant characteristics, sample size, form and dosage of ginger, control intervention, assessment of adherence, outcome measures, methods for statistical analysis, study findings, and adverse events reported. For the outcome data, we extracted the sample sizes of the ginger and control groups, the mean values and standard deviations for continuous outcomes, and frequencies for ordinal outcomes. Where missing information was detected or clarification was needed, we attempted to contact the authors of the primary studies via email.

The Cochrane Collaboration's tool was used for assessing risk of bias in individual studies [17]. This tool allows for assessing risk of bias for individual domains including random sequence generation, allocation concealment, blinding of participants and personnel, blinding of outcome assessment, incomplete outcome data, selective reporting, and other sources of bias (e.g., bias from contamination, bias from carry-over effect, and bias from conflict of interest). Risk of bias for each domain was judged as “high,” “low,” or “unclear” (when too few details were available to make a judgment of “high” or “low” risk). Two of the reviewers independently assessed risk of bias. Disagreements were resolved by discussion and consensus with assistance from the third reviewer.

Data extracted from the included trials were synthesized narratively. Tabulation was used to juxtapose trial characteristics (i.e., participants, intervention, comparison, and outcome measures) and findings. Patterns across the trials were identified in terms of trial characteristics and study findings. Factors that might have influenced the results were further explored. We rated the overall quality of evidence (high, moderate, low, or very low) using the GRADE approach [19]. Based on this approach, randomized controlled trials without serious limitations are rated as high quality. However, the overall quality of evidence can be downgraded depending on the presence of each of the following factors: high likelihood of bias, indirectness of evidence, imprecision of results, unexplained heterogeneity or inconsistency of results, and publication bias. The quality rating falls by one level for each factor [19].

Where available, we used outcome data from the intent-to-treat analysis to calculate effect sizes. For continuous outcomes, we calculated mean difference (where the same scale was used across studies) or standardized mean difference (where different scales were used across studies) corresponding to Cohen's *d*. For ordinal outcomes, we calculated Cliff's delta [20] and subsequently converted the value to Cohen's *d*. We conducted exploratory meta-analysis using random-effects models [17]. *I* ^2^ was used as the measure of heterogeneity. A funnel plot to assess publication bias was not possible because of the limited number of studies (<10) included in the review [17]. RevMan 5.3 and R software were used for the meta-analyses.

## 3. Results

### 3.1. Description of Studies

**Figure 1:**
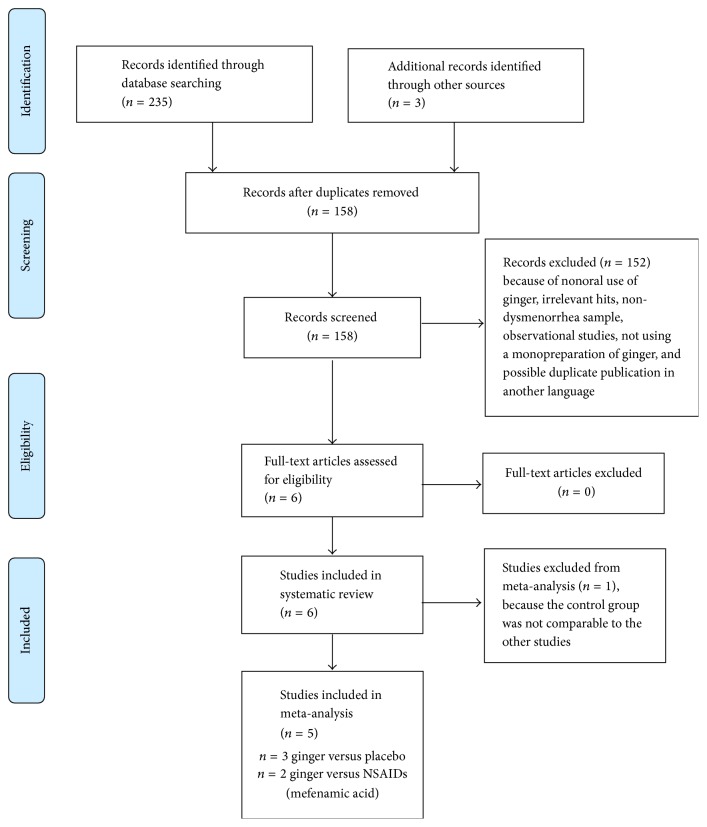
PRISMA flow diagram.

Figure 1 provides a flow diagram of studies identified, screened, included, and excluded for the systematic review and meta-analysis. Six trials met criteria and were included in the systematic review. All the trials were identified through database searching; no relevant unpublished trials or grey literature were identified. We found one paper published in Persian [21] that, with translation assistance, was determined to very likely be a preliminary report of one of the included studies (published in English) [15]. Therefore, the Persian study was excluded to avoid possible duplication of data.  Table 1 summarizes key characteristics of each study.

All included studies were of parallel design. Among the six studies, five were conducted in Iran [14, 15, 22–24] and one in India [25]. Participants were either college or high school students. Five of the six studies included only women with moderate to severe symptoms [14, 15, 22–24]. Four studies specified the inclusion of women with primary dysmenorrhea only, excluding women with secondary dysmenorrhea [14, 15, 23, 24]; however, it is unclear how secondary dysmenorrhea was defined/diagnosed. Across the studies, the sample sizes of the ginger group ranged from *N* = 25 to *N* = 61.

All included trials tested ginger in the form of crude dry powder. It was mentioned in three studies that ginger was processed in an Iranian manufacturing plant [14, 15, 23], but the exact origin of the ginger is not clear across the studies. The quantity of active constituents of ginger was neither tested nor reported in any of the studies. The daily dose of powdered ginger ranged from 750 mg to 2,000 mg. The most common duration and timing of ginger treatment was three days (the first three days of menstruation) [14, 22, 25]. Rahnama et al. tested a five-day regimen (starting two days before menstruation) followed by a three-day regimen (starting the first day of the subsequent menstruation) within the same group of participants [15]. Kashefi et al. used a four-day regimen starting the day before menstruation [24], and Shirvani et al. [23] tested an individualized regimen in which participants were asked to take ginger capsules daily till their menstrual pain was relieved. Participants were typically given ginger and followed for only one cycle, Kashefi et al. being the only exception with ginger being given for two cycles [24]. Adherence to treatment was evaluated only in one study, in which participants were asked to report the number of capsules they took [14]. Ginger was compared with placebo in three trials, two two-arm trials involving ginger and placebo [15, 22] and one three-arm trial involving ginger, placebo, and zinc [24]. Two trials compared ginger with NSAIDs [14, 23]. More specifically, ginger was compared with mefenamic acid in a two-arm trial [23] and with mefenamic acid and ibuprofen in a three-arm trial [14]. Halder conducted a three-arm trial comparing ginger with progressive muscle relaxation (PMR) and an unspecified control condition.

To measure pain severity, four studies [15, 22–24] used the visual analogue scale (VAS), a continuous numerical scale comprised of a 10 cm line. Two studies measured pain severity using ordinal scales: Ozgoli et al. measured pain severity using 4-point verbal multidimensional scoring system (VMS) [14]; Halder measured symptoms severity on a 5-point Likert scale with little information about the scale being reported [25]. Only Halder reported severity of pain at more than one location (e.g., low abdomen and low back) as well as nonpain symptoms (e.g., nausea, vomiting, and diarrhea) using a 5-point Likert scale. Pain duration was assessed in two studies. In Shirvani et al. [23], “days in pain” data were collected; in Rahnama et al. [15], “hours in pain” data were collected. Timing of outcome assessment varied. In two trials [24, 25], participants were asked to rate their symptom severity every 24 hours. In Shirvani et al. [23], symptom severity was rated retrospectively on the last day of menstruation. In the remaining three studies [14, 15, 22], specific timing for outcome assessment was unclear.

### 3.2. Risk of Bias in the Included Studies

**Figure 2:**
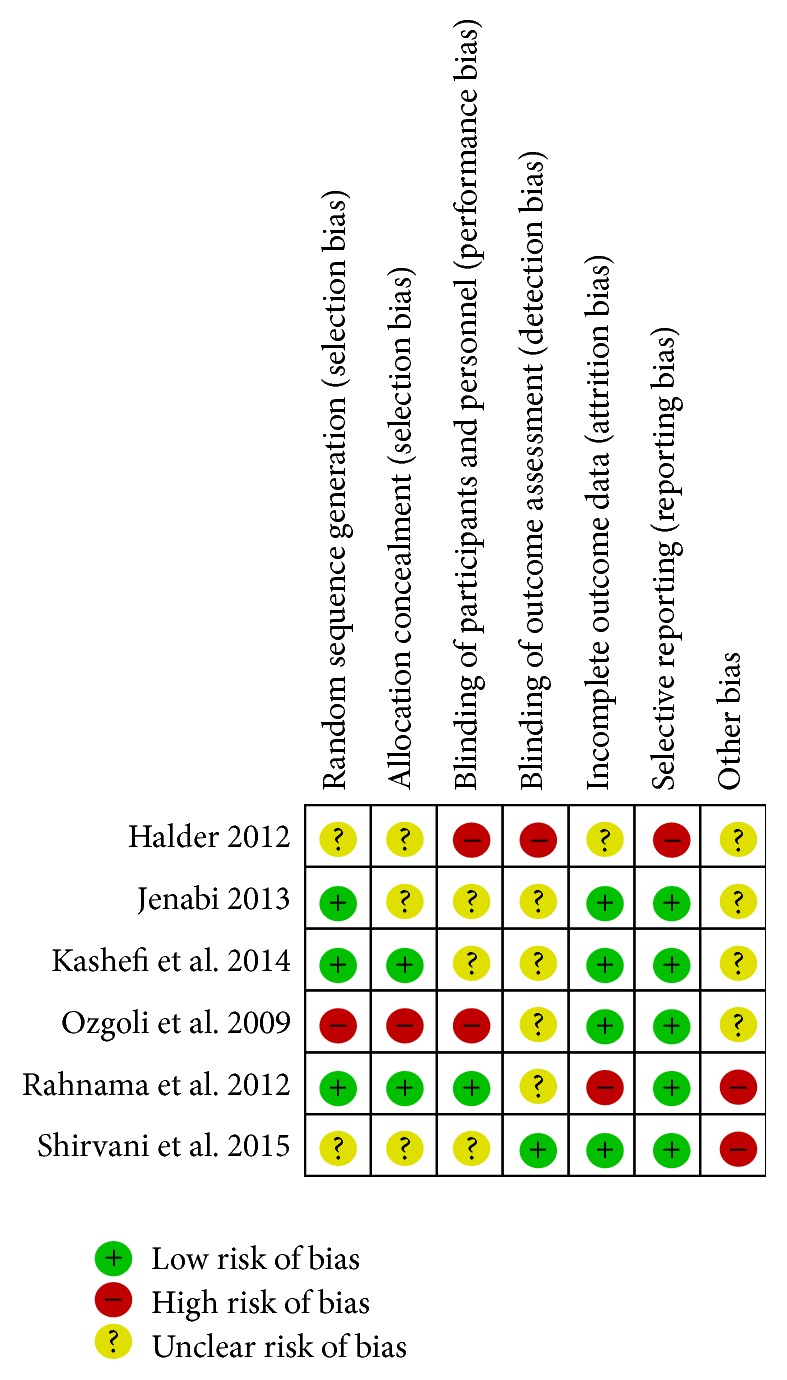
Risk of bias summary: review authors' judgments about each risk of bias domain for each included study.

Figure 2 summarizes the risk of bias for each study. For random sequence generation, three included studies were judged as having a low risk of bias. In each of these three trials [15, 22, 24], it was reported that a table of random numbers was used. Ozgoli et al. [14] were judged as being at high risk ofbias because an alternate assignment approach was used. In the remaining two studies [23, 25], random assignment was stated but the authors gave no detail on the sequence generation.

Allocation concealment was judged as low risk in two studies: In Rahnama et al. [15], allocation lists were managed centrally by a midwife. Kashefi et al. [24] stated that the capsules were identical in appearance and coded by the pharmacologist. Ozgoli et al. [14] was rated at high risk of bias because participants were alternatively allocated into the groups. In the remaining three studies [22, 23, 25], there was insufficient information to assess allocation concealment.

Only one trial was double-blinded (participants and personnel) [15], where packets were coded and capsules were described as identical in appearance, color, smell, and taste. Two trials had a high risk of bias: In one trial [14], ginger and NSAIDs were produced by different pharmacological companies, which made it possible to identify pills. In the other trial [25], blinding was not possible because of the different types of interventions used (i.e., ginger versus PMR). Information was insufficient to assess blinding participants and personnel in the remaining three studies [22–24].

Attrition was acceptable in four studies: There was no attrition in Shirvani et al. [23], Jenabi [22], and Ozgoli et al. [14], and the attrition was low and balanced across groups (5.3% and 6.5%) in Kashefi et al. [24]. In Rahnama et al. [15], however, there was differential attrition, with 22% dropping out of the placebo group and 0% dropping out of the ginger group. In addition, intent-to-treat analysis was not used in this study, which caused high risk of bias related to incomplete outcome data. Information about attrition was unclear in Halder [25].

For bias related to selective reporting, none of the trials was prospectively registered in the trial registries that we searched, which precluded us from comparing a protocol with a published report. As an alternative, we compared the outcomes listed in the purpose statement or methods section with the reported results. We judged reporting bias as low for all studies except for Halder [25] in which the severities of some symptoms were identified in the purpose statement, but not reported in the results.

“Other risk of bias” was judged as high in two studies. In Shirvani et al. [23], there was differential use of extra analgesics between the ginger group and the NSAID group with higher usage in the ginger group. In Rahnama et al. [15], the effects observed for Protocol 2 (three-day treatment) were biased by always being preceded by effects of Protocol 1 (five-day treatment) (i.e., potential carry-over effect). In addition, there was a high risk of contamination between groups since the participants were from the same dormitory. For the rest of the studies [14, 22, 24, 25], “other risk of bias” was unclear due to lack of information. None of the trials declared sources of funding or conflicts of interest.

### 3.3. Effects of Interventions

**Figure 3:**
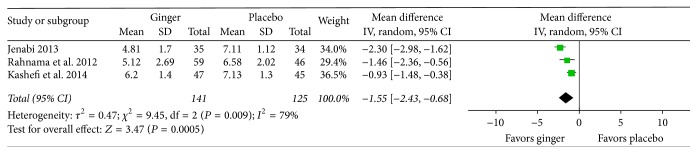
Forest plot for ginger versus placebo, pain severity (10 cm VAS).

All included studies reported beneficial effects of ginger. For three of the studies comparing ginger with placebo [15, 22, 24], ginger was reported to be more effective than placebo in reducing pain severity (see Figure 3 for effect sizes). In addition, one study reported that pain duration (assessed by hours in pain) was significantly reduced with a five-day ginger regimen compared to placebo [15]. For two studies comparing ginger with NSAIDs, the researchers found no significant difference in pain severity between ginger and NSAIDs: Ozgoli et al. [14] reported no significant difference between ginger, mefenamic acid, and ibuprofen; Shirvani et al. [23] reported no significant difference between ginger and mefenamic acid (see Figure 3 for effect sizes). In the single study that evaluated pain duration, no significance difference between ginger and mefenamic acid was reported [23]. Halder [25] compared ginger with PMR and control, finding that both ginger and PMR reduced symptoms of dysmenorrhea, but ginger was more effective in terms of reducing cramping and colicky pain, lower abdominal pain, nausea, and diarrhea.

Two trials [15, 24] reported side effects of ginger. Heartburn was reported in both studies, with occurrence rates of 2.1% and 5.1% [15, 24], and headache was reported in one study, with occurrence rates of 2.1 and 2.2% across two menstrual cycles [24]. No significant difference was found between the groups with respect to side effects [24].

We conducted exploratory meta-analyses on three studies that compared ginger with placebo and on two studies that compared ginger with mefenamic acid (an NSAID). Data were excluded from the meta-analyses in cases where no other study compared ginger with a specific comparison group (PMR [25], zinc [24], ibuprofen [14]), where the control condition was inadequately described [25], or where the data were potentially biased by a previous treatment cycle or protocol (i.e., potential carry-over effect) [15, 24].

**Figure 4:**
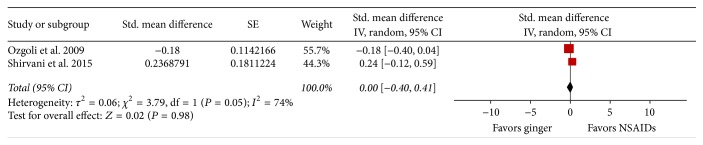
Forest plot of ginger versus NSAID (specifically mefenamic acid), pain severity.

Results of meta-analysis suggest that ginger was more effective for reducing pain severity than placebo (Figure 4). The weighted mean difference on the VAS scale was 1.55 cm (favoring ginger) (95% CI 0.68 to 2.43). Results of meta-analysis for ginger versus mefenamic acid failed to show any difference in pain severity between the two treatments (Figure 4). The standardized mean difference was 0 (95% CI −0.40 to 0.41). Statistical heterogeneity between studies was very high based on the inconsistency index (*I* ^2^ = 79% for ginger versus placebo, *I* ^2^ = 74% for ginger versus NSAIDs). We were unable to conduct meta-analyses on any of the secondary outcomes that we specified in our protocol (i.e., other dimensions of pain and other dysmenorrhea symptoms) because of insufficient data.

## 4. Discussion

This review summarizes evidence from six clinical trials evaluating the efficacy of oral ginger for dysmenorrhea. The available data suggest a promising pattern of oral ginger as a potentially effective treatment for pain in dysmenorrhea. Overall, ginger was reported as more effective for pain relief than placebo, and no significant difference was found between ginger and NSAIDs. These findings, however, need to be interpreted with great caution due to the small number of studies, poor methodological quality, and high heterogeneity across the trials. Based on the GRADE framework to assess cumulative evidence [19], we judged the overall quality of evidence as “low.” We downgraded the quality of evidence from high to low because of the likelihood of bias and unexplained heterogeneity. In addition to the issue of internal validity, external validity of the evidence is a concern. All the included trials were conducted in Asia. Pharmacogenetics and outcome expectancy regarding ginger intervention could differ across cultures and ethnicities, and therefore, it is uncertain whether the results can be generalized to women worldwide. Research conducted in Western countries (US and Denmark) supports the analgesic effect of ginger for arthritis pain [26] and muscle pain after exercise [27]. Presumably, ginger may have analgesic effect among Western women. However, in the context of dysmenorrhea, this premise should be further tested.

In terms of safety, the included trials suggest that ginger is relatively safe, with reported side effects (heartburn and headache) being infrequent and the numbers of adverse events similar for ginger and placebo groups [15, 24]. This is consistent with previous report that ginger has a good safety profile when used appropriately [9, 12, 13]. One systematic review suggests that, as pain treatment, ginger has a superior safety profile to NSAIDs, indicated by fewer gastrointestinal side effects and renal risks [13]. Given the safety profile and preliminary evidence of efficacy, ginger may be appropriate for women with dysmenorrhea who cannot or prefer not to use conventional medications. Patient values and preferences would play a crucial role in treatment decision making. Ginger, however, needs to be used with caution for people who take Nifedipine and anticoagulants due to potential drug interactions [9].

The findings of the current review are consistent with two previous reviews of ginger for pain, which concluded that there is preliminary support of the efficacy of ginger on pain conditions such as osteoarthritis and dysmenorrhea [12, 13]. Findings are also similar, but less positive than those of a recent review by Daily et al. [16]. Daily et al. report a more favorable conclusion regarding the efficacy ginger for dysmenorrhea than we describe here. According to Daily et al. [16], the included trials had “low or moderate” risk of bias and that ginger is “highly effective for treating dysmenorrhea” (p. 2252). In contrast, we gave more “high risk of bias” ratings for included trials. For example, we followed Cochrane Handbook recommendations [17] and judged “alternate assignment” as high risk of bias for allocation concealment, whereas Daily et al. did not. The effect size for ginger versus placebo from Daily et al. [16] was larger than that in our review (~2.2 versus 1.55 on a 10 point scale). This is because, for trials with two-cycle treatment, Daily et al. extracted data from Cycle 2, but we extracted data from Cycle 1. We did that to prevent potential carry-over effect and to make included trials more comparable to each other. Our review also differs from Daily et al. in other significant ways. We prospectively registered the review protocol to increase transparency and safeguard against selective reporting [17]. In addition, our meta-analysis compares ginger not only with placebo, but also with NSAIDs. Given the wide use of NSAIDs among women with dysmenorrhea, the information about comparative efficacy may be useful for researchers and clinicians. Finally, authorship of the Daily et al. article included the president of a dietary supplement company that manufactures ginger supplements, which present the potential for bias.

The current review has several limitations. First, though we believe our search strategy was systematic and comprehensive (not limited to papers published in English), we may have missed relevant trials that were only accessible through non-English databases. Second, the meta-analyses were by nature exploratory, serving to provide a crude overview of the overall direction and magnitude of the results. Because of the high heterogeneity among the studies, results of our meta-analysis must be interpreted with caution. Third, given the limited number of studies and limited quality of the included trials, it was not possible for us to explore sources of heterogeneity through subgroup analysis (e.g., subgroup analysis of high quality studies) or metaregression (e.g., to evaluate dose-response relationships).

There is a strong need to improve the methodological quality of future trials. Future trials need to use appropriate methods for random sequence generation, allocation concealment, blinding participants and personnel, blinding outcome assessors, addressing incomplete data (including using intent-to-treat analysis), and reporting prespecified outcomes. Effective blinding is particularly important in designing future trials. Ginger has a distinct aroma and taste, which poses a major methodological challenge in blinding. If blinding is ineffective, the observed effects may be inflated due to positive expectations. Because none of the trials assessed the success of blinding, it is not possible to ascertain if blinding was effective. Research has shown that participants receiving bottles with ginger capsules could correctly identify the bottle 75% of the time; however, when participants received ginger capsules without containers, they could no longer differentiate between ginger and placebo [28]. A subsequent study demonstrated that ginger could be effectively blinded by using blister packs which minimize the aroma [29]. We recommend that future researchers follow this approach and, at the same time, systematically assess the effectiveness of blinding. In addition, future trials should carefully address potential confounding variables (e.g., use of other analgesics and hormonal contraceptives) through study design or analysis. Future trials need to be appropriately powered. Although power analysis was stated in four trials [14, 15, 22, 23], none of them specified the effect size that was used for calculation. In addition, for the two trials comparing ginger and NSAIDs [14, 23], it was problematic to conclude that ginger was as effective as NSAIDs, because neither trial was designed as an equivalence or noninferiority trial. Future researchers comparing ginger with NSAIDs are encouraged to design noninferiority trials and to power the study with a specified noninferiority margin.

Future publications need to fully describe the methods of trials especially regarding key aspects of the design and ginger preparations. None of the available trials quantified and/or reported the amount of ingredients in ginger preparations. Without such information, it is challenging to make between-study comparisons and clinical recommendations. We urge researchers to consult the CONSORT statement specific to herbal medicine trials [30] when designing and reporting trials. Meanwhile, to allow assessment of publication bias and outcome reporting bias, prospectively registering trials is recommended.

This review suggests potential benefits of oral ginger in managing dysmenorrhea pain; however, the findings need to be interpreted with caution due to the shortcomings in available trials and the exploratory nature of our meta-analysis. Future trials need to be rigorous in design and delivery, with adequate reporting of trial details to enable appraisal and interpretation of results.

## Figures and Tables

**Table 1:** Study characteristics.

Study	Participant characteristics	Sample size		Ginger preparation and dose	Comparison	Outcome measure	Author conclusions	Side effects
		Ginger group	Control group					
Jenabi, 2013 [22]	(i) College students in Iran (ii) Having moderate to severe dysmenorrhea	35	34	Capsule of ginger powder (unknown origin and constituents) 500 mg TID × 3 days (first 3 days of a period)	Placebo	Pain severity (VAS)	(i) The mean change in pain severity in ginger group was significantly greater than the placebo group.	None reported

Rahnama et al., 2012 [15]	(i) College students (>18 yo) in Iran living in the dorms (ii) With primary dysmenorrhea (iii) Having moderate to severe dysmenorrhea (iv) BMI between 19 and 25 (v) Not on OCPs	59	46	Capsule of ginger powder which was dried in a dark condition at room temperature (processed in Iran, unknown constituents) Protocol 1: 50 mg TID × 5 days (2 days prior to day 3 of period) Protocol 2: 50 mg TID × 3 days (first 3 days of a period)	Placebo	Pain severity (VAS) Pain duration (hours in pain)	(i) Pain severity was significantly reduced with ginger compared to placebo for both Protocol 1 and Protocol 2. (ii) Pain duration was significantly reduced with ginger compared to placebo for Protocol 1 but not for Protocol 2.	GI side effects were reported in 5.1% of ginger group (heartburn) and 8.7% of the placebo group (nausea)

Kashefi et al., 2014 [24]	(i) High school students (15–18 yo) in Iran (ii) Moderate to severe dysmenorrhea (iii) With primary dysmenorrhea (iv) Not on OCPs, hormonal meds, or analgesics	47	45 Placebo 54 Zinc	Capsule of ginger powder (unknown origin and constituents) 250 mg TID × 4 days for 2 menstrual cycles	Placebo Zinc 220 mg 3 times/day for 4 days	Pain severity (VAS)	(i) Compared with the placebo group, the ginger group and zinc group reported more symptom improvement for both Cycle 1 and Cycle 2.	Ginger group reported headache (2.1% in Cycle 1, 2.2% in Cycle 2) and heartburn (2.1% in Cycle 1 and 4.4% in Cycle 2) No significant difference in adverse effects among the groups

Ozgoli et al., 2009 [14]	(i) College students (>18 yo) in Iran living in the dorm (ii) Having moderate to severe dysmenorrhea (iii) With primary dysmenorrhea (iv) Not on OCPs (v) BMI between 19 and 36	50	50 Ibuprofen 50 Mefenamic acid	Capsule of ginger powder (Zintoma an Iranian brand, unknown constituents) 250 mg QID × 3 days	NSAIDS Ibuprofen 400 mg 4 times day for 3 days Mefenamic acid 250 mg 4 times day for 3 days	Pain severity assessed by the VMS	(i) No significant difference in pain severity was found between ginger, ibuprofen, and mefenamic acid.	None reported

Shirvani et al., 2015 [23]	(i) College students (>18 yo) in Iran living in the dorms (ii) With primary dysmenorrhea (iii) Having moderate to severe dysmenorrhea (iv) Not using IUD or OCs	61	61	Capsule of ginger powder (Zintoma, an Iranian brand with unknown constituents) 250 mg QID until pain relieved	Mefenamic acid 250 mg 3 times per day until pain was relieved	Worst pain severity assessed by VAS Pain duration (days in pain)	(i) No significant difference in pain severity was found between ginger and mefenamic (ii) No significant difference in pain duration was found between ginger and mefenamic acid.	None reported

Halder, 2012 [25]	(i) Nursing students in India (ii) With primary or secondary dysmenorrhea (iii) Not on IUD or taking OCPs	25	25 PMR 25 Control group	Capsule of ginger powder (unknown origin and constituents) 1000 mg BID × 3 days	PMR once/day × 3 days Control (little information provided)	Severity of dysmenorrhea symptoms (five point scale)	(i) Both ginger and PMR were more effective than control in managing dysmenorrhea symptoms, but ginger was more effective than PMR for cramping, colicky pain in lower abdominal pain, nausea, and diarrhea.	None reported

TID: three times a day.

VAS: visual analogue scale (0–10 cm).

yo: years old.

BMI: body mass index.

GI: gastrointestinal.

OCPs: oral contraceptive pills.

QID: four times a day.

NSAIDs: nonsteroidal anti-inflammatory drug.

VMS: verbal multidimensional scoring system.

IUD: intrauterine device.

PMR: progressive muscle relaxation.

BID: twice a day.
